# Recovery After Psychosis: Qualitative Study of Service User Experiences of Lived Experience Videos on a Recovery-Oriented Website

**DOI:** 10.2196/mental.9934

**Published:** 2018-05-08

**Authors:** Anne Williams, Ellie Fossey, John Farhall, Fiona Foley, Neil Thomas

**Affiliations:** ^1^ School of Health Sciences Faculty of Health, Arts and Design Swinburne University of Technology Hawthorn, Melbourne Australia; ^2^ Living with a Disability Research Centre La Trobe University Melbourne Australia; ^3^ Department of Occupational Therapy School of Primary and Allied Health Care Monash University Melbourne Australia; ^4^ Department of Psychology and Counselling La Trobe University Melbourne Australia; ^5^ NorthWestern Mental Health Melbourne Health Melbourne Australia; ^6^ Centre for Mental Health Swinburne University of Technology Melbourne Australia; ^7^ Monash Alfred Psychiatry Research Centre Alfred Hospital and Monash University Central Clinical School Melbourne Australia

**Keywords:** mental health recovery, telemedicine, mental health services, psychotic disorders, schizophrenia, qualitative research

## Abstract

**Background:**

Digital interventions offer an innovative way to make the experiences of people living with mental illness available to others. As part of the Self-Management And Recovery Technology (SMART) research program on the use of digital resources in mental health services, an interactive website was developed including videos of people with lived experience of mental illness discussing their recovery. These peer videos were designed to be watched on a tablet device with a mental health worker, or independently.

**Objective:**

Our aim was to explore how service users experienced viewing the lived experience videos on this interactive website, as well as its influence on their recovery journey.

**Methods:**

In total, 36 service users with experience of using the website participated in individual semistructured qualitative interviews. All participants had experience of psychosis. Data analysis occurred alongside data collection, following principles of constructivist grounded theory methodology.

**Results:**

According to participants, engaging with lived experience videos was a pivotal experience of using the website. Participants engaged with peers through choosing and watching the videos and reflecting on their own experience in discussions that opened up with a mental health worker. Benefits of seeing others talking about their experience included “being inspired,” “knowing I’m not alone,” and “believing recovery is possible.” Experiences of watching the videos were influenced by the participants’ intrapersonal context, particularly their ways of coping with life and use of technology. The interpersonal context of watching the videos with a worker, who guided website use and facilitated reflection, enriched the experience.

**Conclusions:**

Engaging with lived experience videos was powerful for participants, contributing to their feeling connected and hopeful. Making websites with lived experience video content available to service users and mental health workers demonstrates strong potential to support service users’ recovery.

## Introduction

Experiences of psychosis have the potential to be prolonged over many years and to significantly disrupt social, emotional, occupational, and financial pathways through life [1]. Early experiences of psychosis often occur during adolescence to early adulthood, which regardless of illness course can lead to persistent social disadvantage [2]. Close relationships, socializing with others, and employment can be difficult to maintain [2-5]. Approximately 23-33% [1,2] of people with psychotic disorders also experience ongoing symptoms and disability, typically leading to continued engagement with mental health services [1].

Personal recovery—a process of rebuilding a meaningful life in the context of living with a mental illness—has become an influential way of understanding how people manage their lives after experiencing psychosis [3,5-7]. This understanding has arisen primarily from people’s own accounts of recovery and is often contrasted with recovery being framed in terms of symptom remission [3]. Based on an extensive narrative review and synthesis of literature about recovery, Leamy et al [8] summarize recovery as being an active, individual, and nonlinear journey that may occur in phases and is influenced by five recovery processes: Connectedness, Hope, Identity, Meaning, and Empowerment (CHIME). Recovery-oriented practices that support these processes are now expected of mental health services and practitioners working with service users [9-11]. Ways of promoting recovery may include supporting service users to self-manage their mental health [12], offering peer support [13], and fostering recovery-promoting relationships with mental health workers [14].

Using contemporary technologies and the Internet to support recovery [15-18] and self-management [19] among people experiencing psychosis is a relatively recent phenomenon. The Internet, coupled with the widespread uptake of mobile devices, has the potential to increase access to and use of evidence-based resources that can empower users in self-management [16]. Digital health interventions are feasible and acceptable among people experiencing psychosis [20-22], although (as with other populations) engagement over time can be difficult to sustain [15,19] and the cost of continuing Internet access may be prohibitive [17]. Naslund et al [23] contend that peer-to-peer support gained through self-forming, online communities on social media sites such as Facebook and Twitter could be the future of mental health care. However, they also outline drawbacks, including the potential for unreliable online information, unhelpful online relationships, and difficulty transferring learning into the offline environment.

Incorporating digital health interventions and peer support into existing mental health services presents a way forward. Strand et al’s [24] recent scoping review asserted that “e-recovery” can “potentially facilitate recovery-oriented care” (p. 11) in mental health services. Peer support was central to five of six interventions included in their review, of which two involved experience-sharing with peers through forums, stories, or videos [25,26]. These included a Finnish Web-based psychoeducation program undertaken by inpatients with the support of psychiatric nurses [25], and a website used to facilitate shared decision-making about medication prescription by outpatients with the support of peer workers in the United States [26]. However, in the context of rapidly developing technology and the wide reach of the Internet, there have been surprisingly few advancements in integrating recovery-focused digital health interventions into mental health services. This study reports on service users’ experience of an innovative Web-based resource with potential to address this gap.

The Self-Management And Recovery Technology (SMART) research program has involved the development of an interactive website of recovery-oriented resources primarily based on videos of people with lived experience of psychosis, communicating how they have navigated issues in their own recovery [27]. Content was co-designed with people with experience of mental illness and founded on the conceptual framework of the CHIME recovery processes [8]. A range of potential applications of this digital technology were envisioned [27,28]. The website was designed to be used both as a therapeutic tool for workers and service users to access together on a tablet computer during appointments and to be accessible directly by service users from their own devices.

As part of the overall SMART research program led by author NT, qualitative research aimed to explore how service users experienced using this interactive website and how this influenced their recovery journey. This paper focuses specifically on service users’ experiences of viewing the lived experience videos on the website, as this was identified as a pivotal element of the overall experience.

## Methods

### Research Design

This study was guided by constructivist grounded theory (CGT) [29], with a focus on actions and social processes occurring within a specific context. This methodology was selected as attending to social processes [29] corresponded with introducing an Internet-based resource into an inherently interactive relationship between a service user and mental health worker in community mental health services [30]. We were interested in exploring how both parties experienced using the Internet-based resources and how the experience influenced their interactions. CGT involves an iterative process of collecting rich empirical data with concurrent data analysis, followed by further data collection in areas of progressively more specific conceptual interest [29]. This paper portrays one of the areas of conceptual interest identified in the study, that is, the service users’ experiences of the lived experience videos. Furthermore, the CGT method recognizes that the research process involves a shared construction of meaning between participants and researchers [29]. People with lived experience of psychosis who were members of a reference group for the SMART research program contributed to the development of the research design and data collection tool. The emerging themes were shared with participants and others with lived experience to seek their feedback on the interpretation of the data.

### Recruitment and Setting

This qualitative study was conducted in two Australian clinical community mental health services that provide psychiatric treatment and recovery-oriented services and four nonclinical community mental health services that provide psychosocial rehabilitation and recovery support [4]. Eligible service users were over 18 years of age, with a diagnosis of a psychotic disorder, and had sufficient English proficiency to participate in an interview. They had either completed use of the SMART website with a mental health worker employed by the SMART research program or had used the website for at least 3 months with their usual mental health worker (both are hereafter abbreviated to workers). Workers came from disciplines including psychiatric nursing, social work, occupational therapy, psychology, and community work. Invitations to participate in this qualitative study were sent to SMART research program participants, who met these criteria and had agreed to receive invitations about SMART-related studies, by mail or email. A participant information and consent form was subsequently sent to those who were interested. In total, 36 service users (hereafter referred to as participants) consented to participate. They were predominantly women (24/36, 67%), had a mean age of 41 years (range 19-64 years), and described having experienced illnesses such as schizophrenia, bipolar disorder, and psychotic depression, most often with a duration of more than 10 years (Table 1). All were living in the community, in predominantly urban areas, and receiving one of, or both clinical and nonclinical community mental health services. Participants rated themselves as confident using computers and the Internet (average self-rating of 4.2/5 where 5=highly confident); 58% (21/36) of participants had access to more than one device to connect to the Internet; and 61% (22/36) used the Internet “sometimes” or “often” for understanding or managing their health (Table 1).

### Digital Health Intervention

**Figure 1:**
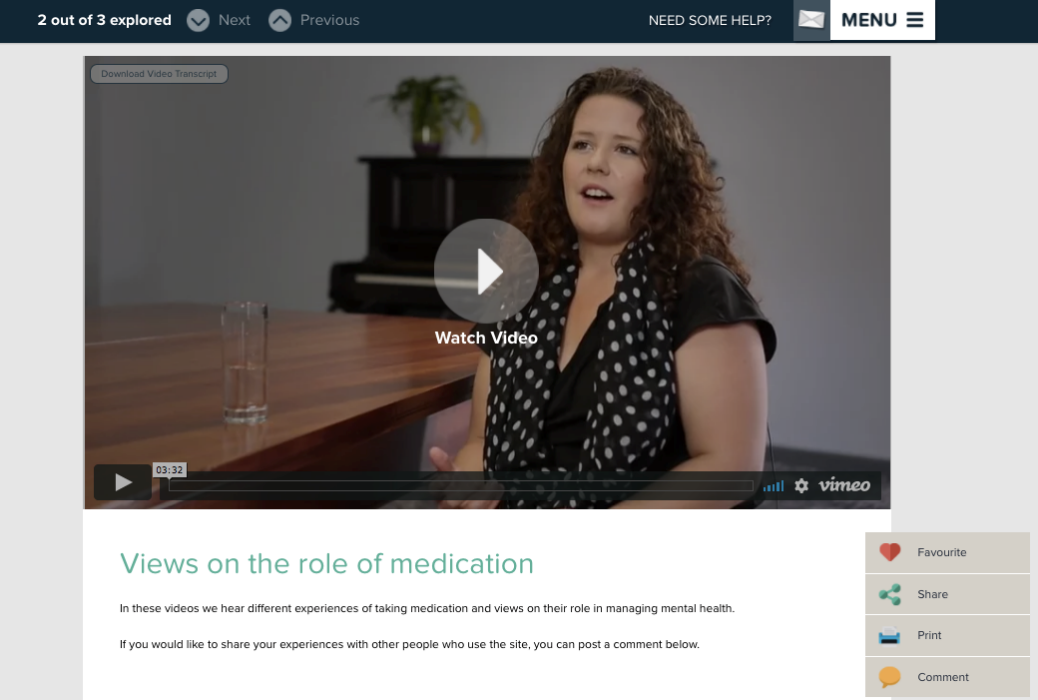
Sample image of lived experience video.

The SMART interactive website aimed to promote recovery, be accessible to people experiencing psychosis, and enable optimal use on a tablet computer or mobile phone [27]. It includes material on recovery (introduction and promoting hope), managing stress (common stressors and coping strategies), health (self-managing physical health, medication, diet, and sleep), me (identity and stigma from a strengths approach), relationships (interpersonal relationships and mental health), empowerment (interactions with mental health services, rights and advocacy), and life (meaning, values, and goals in life). The website has 37 videos embedded throughout of 2-3 minutes length, featuring 7 male and 4 female peers (full details in [27]). Of these, 11 videos introduce each of the peers, who are of diverse age, ethnicity, employment status, and sexuality [27], and 26 videos show 4-6 peers talking about their experiences, views, and actions in relation to specified topics, for example, “experiences of making changes” (health topic), “identifying our strengths” (me topic), and “views on getting the most out of services” (empowerment topic; Figure 1). Participants can post their response to questions that follow videos using an online name, read comments written by others, and save videos to a “personal favorites” area. The website also includes a peer discussion forum open to users with lived experience only, tools for charting personal experiences (eg, stress or mood), additional videos of peer leaders who introduce the topics, videos of clinicians and academics discussing topics such as “making plans and taking action” (life topic), and links to other evidence-based websites.

In this qualitative study, 26 participants interviewed used the SMART website with a trained worker employed in the research program, and 10 used the website with their usual worker at the mental health service they attended. The website was available for use in individual meetings occurring in mental health services or in participants’ homes. All participants were given a personal login in the first meeting and could use the website between meetings if they had access to an Internet-enabled device. Use with a trained worker entailed up to eight 50-minute meetings integrating use of the website, held over a 3-month period. Website use with a usual worker occurred in routine meetings for up to 6 months, with flexibility regarding when the website was used alongside other usual work undertaken by the pair. Routine meeting frequency varied considerably from weekly to monthly, as did the length of these meetings, from approximately half an hour to 2 hours.

Workers brought an Internet-connected tablet computer to meetings, to facilitate access to the website. Participants logged into the website, were initially introduced to the first topic called “recovery,” and could then explore the application as they chose. The workers provided technological and navigational support and guidance and discussed the content with the participant. Participants explored topics in the website at their own pace, with some moving through a new topic in each meeting and others choosing to concentrate on fewer topics. Despite this variation, all topics included peer-based content. Those who used the website between meetings using their personal devices could review videos, use tools to monitor their experiences, record personal goals or values, or communicate with peers through the forum. All participants continued to receive their usual clinical, rehabilitation, and support services throughout the research and had ongoing access to the website once the research period ended.

### Data Collection

Data were collected in individual interviews conducted by the first author, who had no prior relationship with any of the participants. A semistructured interview guide with broad questions enabled exploring the overall experience of using the website resources (see Multimedia Appendix 1). Open-ended questions were pre-determined yet flexible [31], to enable following up responses in subsequent interviews [29]. Interviews occurred shortly after participants had completed their sessions with the trained worker, or after having website access for at least 3 months for participants who used SMART with their usual worker.

**Table 1:** Participant characteristics (N=36).

Characteristics		Participants, n (%)
**Website use**		
	Used with trained mental health worker for 8 sessions	26 (72)
	Used with usual mental health worker for up to 6 months	10 (28)
	Used with worker only	12 (33)
	Used between sessions with worker	24 (67)
**Gender**		
	Female	24 (67)
	Male	12 (33)
**Age, years**		
	<30	7 (19)
	30-45	14 (39)
	>45	15 (42)
**Experience of mental health issues, years**		
	1-5	5 (14)
	5-10	5 (14)
	>10	26 (72)
**Income**		
	Disability support pension	29 (80)
	Other government allowance	5 (14)
	No government allowance/Unstated	2 (6)
	Part-time employment (in addition to allowance)	6 (17)
**Devices used to access the Internet**		
	Own computer	22 (61)
	Own tablet device	13 (36)
	Own mobile phone	24 (67)
	Community or service computer	10 (28)
	No access	1 (3)
**Frequency of Internet use in a typical week**		
	Every day	18 (50)
	Most days	4 (11)
	A few days	5 (14)
	Once or twice	6 (16)
	Not at all	3 (8)
**Typical use of the Internet for health information**		
	Often	6 (17)
	Sometimes	16 (44)
	Rarely	9 (25)
	Never	5 (14)

Interviews occurred face-to-face in mental health services (n=24) or over the telephone (n=12) and lasted on average 41 minutes (range 18-65 minutes). They were audio recorded and transcribed, or handwritten notes were made if the participant preferred. All participants were sent a transcript of their interview. Six service users participated in a second individual interview, 2-3 months after their first interview, to explore their experiences using the SMART website over time. Participants were reimbursed Aus $30 per interview. Researcher reflection occurred alongside data collection to enhance the interviewer’s awareness of self, relationship, process, and content in each interview, as suggested by Mruck and Mey [32].

### Data Analysis

Data analysis followed principles of CGT [29] and occurred concurrently with data collection. The first author completed initial line-by-line coding of interview transcripts using gerunds to identify actions in the data [29]. Concurrent memo-writing allowed reflection on recurring and contrasting codes, which were subsequently checked with the first 8 participants in two follow-up interviews and via an initial newsletter mailed to participants. The importance of the lived experience videos to participants was identified early in the analysis, as indicated by the following excerpt from a memo written after the third interview:

The peer videos seemed to be the most important aspect of the site (to the participant) as she identified emotions that she feels but cannot express and recognized a sense of self-identity that she does not express (outside of her family). Despite the connections being virtual, she felt connected to this greater community of people whose experiences she could relate to.

This lead was followed-up in subsequent interviews. As further interviews were conducted, analysis shifted to include focused coding and category development, as demonstrated for the category “Knowing I’m not alone” in Table 2. Clustering, which involved creating a nonlinear and visual chart to map a central category [29] and analysis using QSR International’s N-Vivo 11 qualitative data analysis software [33] were used to further consider the meaning of the data. The first 3 authors reviewed the evolving analysis in regular meetings. Additional member checking included discussing developing findings twice with a university-hosted panel of people with lived experience of mental illness, a presentation to a conference with an audience including people with lived experience of mental illness, and sharing findings with participants in a second newsletter.

### Ethics and Credibility

Ethical review and approval for the study was obtained from all participating sites and two university human ethics committees. All participants provided informed consent prior to participating. Credibility was enhanced by collecting background data to portray the context in which participants used SMART and by seeking sufficient detailed and multiple accounts of views and actions from participants who varied in age, background, and experiences [29]. Participants were diverse in how much (from minimal to extensive) they used the SMART website and the extent to which they used it by themselves outside of their meetings with a worker.

**Table 2:** Coding examples.

Initial codes	Focused code	Category
Realizing I am not alone Feeling not as alone; Feeling like you’re not alone Feeling connected Feeling like I belong Being part of something Connecting with others going through the same thing Not having to go through it by myself Connecting with peers straight up Knowing you’re not alone Knowing I’m not the only one Realizing I’m not the only one Thinking I was on my own Needing to isolate myself; needing solitude	Realizing I am not alone This code relates to service users realizing that they are not alone. The feeling of not being alone encompasses feeling connected, feeling like I belong, and being part of something. There is implied relief at not having to go through this by myself. This contrasts with prior thinking that I was on my own. One participant discussed how she had needed to isolate herself and needed solitude when unwell: some alone time was necessary.	Knowing I’m not alone Incorporates focused codes Realizing I am not alone Hearing hidden views Feeling exactly the same Hearing and seeing peers talking about their experiences in the website led participants to realize that others shared their experiences and that they were not alone. Participants described feeling connected, belonging, or being part of something after exploring the lived experience content in the website, in contrast to previously “thinking I was on my own”. Knowing I’m not alone was strengthened by “hearing hidden views” that were otherwise not available to participants in the community and “feeling exactly the same” as the peers.

## Results

### Overview

**Figure 2:**
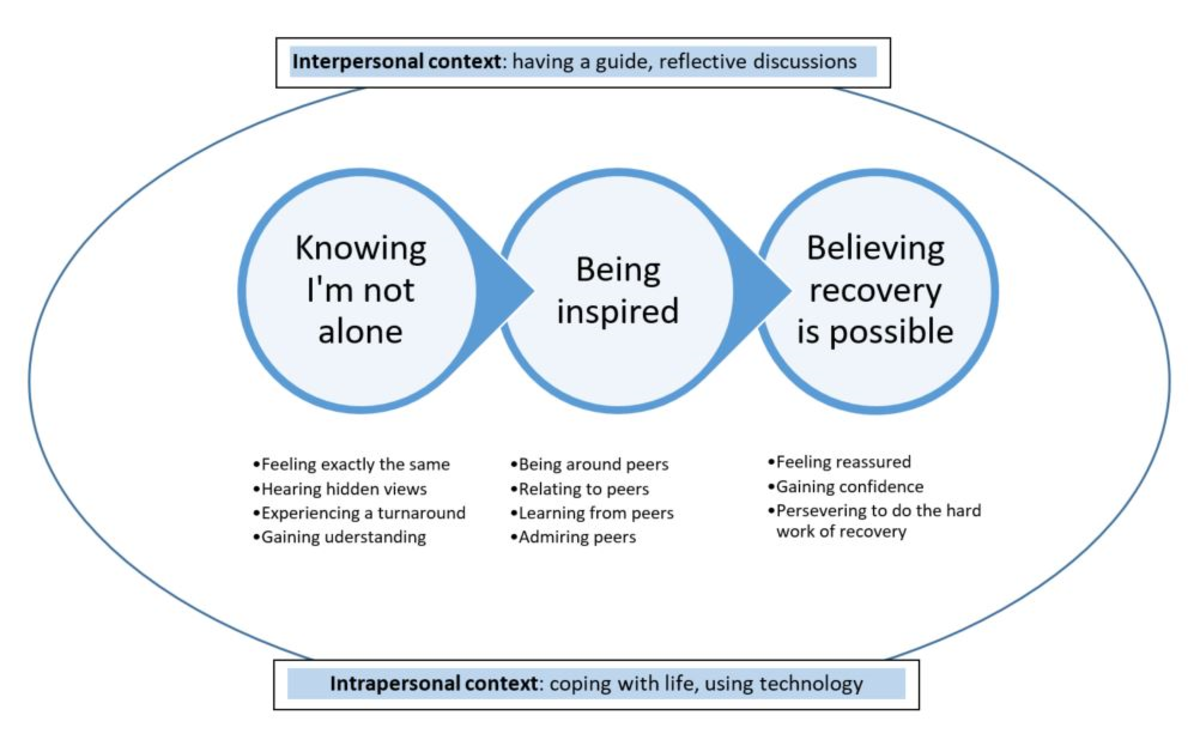
Categories, codes, and contexts related to engaging with lived experience videos.

In total, 36 participants who used the SMART website attended one interview, and 6 of them agreed to a follow-up interview. The analytical process identified that having access to videos of peers speaking about their experience was important to many participants in (1) “knowing I’m not alone,” (2) “being inspired,” and (3) “believing that recovery is possible.” Figure 2 represents these categories and their focused codes and includes contextual factors that influenced participants’ experiences. The intrapersonal context included their prior actions to manage their health and their use of technology. The interpersonal context included using the resources with a worker who guided them and facilitated reflection. Quotes are attributed to participants using their self-selected pseudonyms and are used to illustrate each category and context.

### “Knowing I’m Not Alone”

Participants were quickly drawn into the website when the life circumstances and events discussed by peers in the videos resonated with their own experiences. Participants frequently reported feeling less alone after watching others talking about their experiences, as Reese explained:

I found quite a few of the videos quite helpful, especially when they were saying things that I agreed with, which was really, really cool…made me feel like I wasn’t alone and quite a few of my illnesses, they actually do make you feel quite, quite alone a lot of the time, so for me to be able to see that there were other people out there struggling just as much as I was, or were struggling just as much as I was…was quite helpful.

Participants also discussed feeling connected or belonging after watching and listening to lived experience videos, as Amelia described:

There were other people going through a similar situation and they were telling about their story and then it just seemed like I can relate somehow, and…it just seemed that I’m not the only one with this type of problem, there are other people with other different types of problems, I don’t have to go through it by myself.

Jamin conveyed his sense of personal connection when he stated: “I’d love to meet them sometime because they’re awesome people…I made friends that don’t even know me.”

“Knowing I’m not alone” was strengthened by hearing hidden views that were otherwise not available to participants in the community and “feeling exactly the same” (Suzanne) as the peers. Realizing that others shared their experiences provided a sense of relief and differentiated their engagement from their previous help-seeking, as Amy noted:

I’d never seen that stuff before and stuff that was being said out loud and being recorded, and I was like “wow,” this is stuff that I think in my head, that I’ve come to a conclusion to for being mature in my illness, a 10-year maturity in my illness, but no one’s ever told me it like this.

Connecting with the peers through the videos was initially made possible by the interpersonal context of using the website with the worker who supported participants to “get the hang of it” (Melanie), “remind(ed) me how to find certain things” (Bill), or “prompted me to put something (my views) down” (Kali). As Paul said, “having a person beside you guiding you through the Internet, it’s very handy.” Personal reflection about topics arising in the videos was also enriched by discussions that opened up with workers during the sessions:

What I liked at the beginning of the topics were the peer discussions—“the videos where peers talked about an issue”. They were “really beneficial.” I could identify things that I had done. They began a discussion (between me and worker). “[Worker] helped me apply the information to what mattered.”

Sue

Amy described the value that using the website with a worker added to her experience:

She let me lead the whole thing. So every time I’d see her, “I want to see the SMART website with you because it actually motivates me to do it rather than just doing it at home alone…” When I went on to the website, I said to her, “actually I would like some assistance with this, in reflecting on this topic, because I don’t quite understand it and I do need someone to talk to about it.”

Participants’ intrapersonal context, in particular their own experiences of coping over time, impacted which videos they chose to watch. Experiences discussed by peers could be personally challenging, leading participants to avoid some topics, such as “Empowerment” or “Relationships”. For Kate, it was the “Me” topic:

I would have had trouble with that because I don’t identify with myself all that well. I probably would have got a lot out of the peer videos with that one I think, ‘cos they would have identified themselves, and I would have been able to relate to them.

However, using the website with a worker who invited participants to choose the topics that they wished to explore supported participants to engage with videos addressing difficult topics when they felt ready. The consequences of hearing about challenging experiences from the peers could be transformative, such as Pam described:

I suppose it made me feel less ashamed about having mental illness because even though I have been in the system for quite some time, I still felt quite bad about myself, and just having—a listen to some of the peer workers in what they said about stigma and that, it just really, it felt very empowering…so that was a big turnaround for me.

### “Being Inspired”

The majority of participants who watched the videos in the SMART website also spoke of gaining inspiration, describing the peers as “uplifting” (Awareness), “encouraging” (Julie), “confidence building” (Kali), and “reassuring” (Vlad). As Kali elaborated:

I used it for confidence building and also relate, relate to other…people out there that have schizophrenia or other mental health issues, to feel like I belong…and I found it really reassuring, especially knowing that the people that I was watching that had recorded their stories are now in recovery and they’ve stayed that, some have stayed that way, some are working, it was inspiring stuff.

Learning from the peers, including learning unexpected, new information, or alternatively solidifying existing knowledge, contributed to being inspired. For example, Harley said, “After watching the videos I saw that I was doing things the wrong way and I could try doing things a new way.” Omar valued how “watching the videos did put things in perspective, because they were people who had lived experiences.”

Being inspired by peers included expressing admiration for them and their willingness to share their successes and challenges. Furthermore, reciprocity was possible when participants like Ingrid were “able to relate to their stories, understand them and have empathy.” Jamin also perceived that by adding his comments on the videos he was giving peers “encouragement to keep on doing what they are doing, a great big tick because they were really, really doing it very well.” The diversity of experiences and views shared in the videos provided opportunities for participants to find different points of connection with the peers. For example, participants recognized that differing recovery perspectives and experiences were possible, agreeing with some peers but disagreeing with others. Zara said:

It was reinforcement, which was useful because you sort of think you know stuff but then it sort of solidifies what you know…listening to the different characters that were speaking, just to get their point of view, just to get the broad spectrum of what people experience and how it can be very similar to me or very different to me, or something in between.

Intrapersonal factors, including past experiences of coping with life, influenced how participants felt about the peers and whether or not they found them inspiring. For example, Athalia found watching the videos “draining” and “tiring,” as she felt the need to try to support the peers, as she had done in other Internet-based peer networks. Latte and Guilia wanted to “move on” from focusing on their mental illness and did not want to be connected to others who shared this experience, while Tricia’s experience did not align with what she heard from the peers:

I just couldn’t sort of relate to it at all. Thought, you know sometimes I thought you’re just really lucky, you’re really lucky that things are that way for you because, if things are simple and you think that’s how you can recover like that, but it’s just not my experience.

None of these few participants reported negative consequences from watching peer videos. Instead, after deciding that the videos were not useful for them, they chose to use other resources in the website that supported their self-management. For example, they used charts to monitor their stress, sleep, or diet; watched videos from clinicians; and talked with the worker about topics in the website. Having used the website infrequently with their usual worker, 2 other participants reported insufficient use to gain benefit from the peer videos.

### “Believing that Recovery is Possible”

Videos exploring peers’ perspectives on recovery supported participants to reflect on their understanding of this sometimes-confusing idea, such as Kelly demonstrates: “I didn’t understand what recovery was, but now I understand it’s sort of like getting back to normal…like you know, having positive thoughts and things.” Sylvester experienced a transformation in his understanding:

the one that sticks in my mind, would be about, about having recovery and me thinking that recovery was about, like me trying to be…well, but a traditional well, like thinking that I was going to be, going to get better and not have a mental illness for the rest of my life and then watching, watching different videos on the SMART website and that, and them saying that recovery is a different, you’ve got different terms of recovery. That was a real breakthrough for me.

Participants who felt connected to the peers and inspired by them, listened to the peers’ recovery experiences and believed that their own recovery was possible. Reese said, “Looking at the videos it was, wow, they seem a lot like me and if they can do it, I can too.” Viv’s experience was similar: “I suppose I looked up to these people on the site and thought, well if other people can, with a mental illness, can be articulate and have a voice and have meaning in their life, well that means perhaps I can too.”

The intrapersonal context of having used coping strategies over many years influenced participants’ response to the lived experience videos. Watching a video of a peer saying that recovery is hard work confirmed Pam’s experience and deepened her resolve to persevere. Participants who perceived that they were “on the road to recovery” (Jacqui-Maree) gained confidence that they were indeed doing the right thing and felt able to continue “full speed ahead” (Zara). After completing eight sessions of SMART, Libby, who like others had experienced past trauma, expressed her strengthened determination: “I can see it, I’m going to recover, I want to recover, I have to recover.”

Hearing about actions that peers took in their recovery and discussing these with a worker was empowering and provided participants with new strategies that they could try out in their lives. Bruce stated that, “being able to talk to someone while discussing this sort of stuff, I think it was great… it was making me think about the things that would help you, sort of get you back on your feet.”

Personal access to technology also affected participants’ engagement with the videos beyond meetings with the worker. Being able to hear the peers “over and over” through logging into the site and watching the videos at home supported the information to “sink in” for Viv. Participants who had no or limited Internet access at home due to reception, accommodation, or financial difficulties, did not have this opportunity.

The lived experience videos stood out in the memories of the 6 participants who had a second interview up to 3 months later, even when these participants had not viewed the website for some time. Ingrid described the value of having ongoing access to the videos like this:

To have those videos there to view them, especially at your own viewing time, like it’s private, so you can cry, you can laugh, you can basically go through whatever you want, no one can see what you’re doing, it’s in the privacy of your own home…So there’s no judgement, there’s no judgement of who you are or what you’ve been.

Additionally, in second interviews participants related how what they had seen in the peer videos had influenced their actions. For example, Sue stated:

I remember the “people talking about their experiences” [in the videos]. Some of the issues, like relationships, that are “prominent for me now”. I remember the “medication and negotiating in the mental health system”. I was on a medication at the time that was having terrible side-effects. After I saw different ways you could talk about medication, I realized that they had to listen to my experience. I kept “pushing it” with the doctor, I wouldn’t take no for an answer.

In summary, engaging with peers’ lived experience on the SMART website through videos and discussing the content with a worker prompted reflection on the personal meaning of recovery, provided strategies to support recovery, and generated or affirmed participants’ determination to strive for recovery.

## Discussion

### Principal Findings

This study explored service users’ experiences of using an innovative and interactive recovery-oriented website, SMART [27,28]. Overall the findings show that using the SMART website was viewed as positively supporting participants in their personal recovery journeys. More specifically, watching the videos of people with lived experience of psychosis on the website supported recovery processes by providing relief to service users that they were not alone, inspiring hope, and supporting them to revise and affirm a personal meaning of recovery. These findings provide evidence that from the perspective of service users, watching lived experience videos on a website can contribute to participants feeling less alone and more hopeful as they recognize that they share experiences with other people who they admire. Engaging with lived experience through the videos appeared to provide similar benefits to meeting other people with experience of mental illness, which has been identified as a source of hope [5,34,35] and of acceptance and self-esteem [36] for people with psychosis. This finding extends on the SMART pilot study in which participants felt more connected with people (7 out of 10 participants) and more hopeful (9 out of 10) after using the SMART program [27]. Our findings also have striking similarities with Naslund et al’s [37] analysis of comments made by viewers of lived experience videos uploaded to YouTube, strengthening the evidence that peer support can be experienced through the Internet. Using videos developed with people with lived experience and informed by ideas about recovery [27] overcame the limitations in trustworthiness of videos posted to public Internet sites such as YouTube, as described by Naslund et al [37].

Including lived experience videos in digital health interventions may be particularly helpful for service users who are unable to access person-to-person peer support. For example, in Australia in 2010, only 4.7% of people experiencing psychosis and connected to mental health services reported that they participated in peer-led support groups [4]. Lived experience videos could also be offered to people experiencing psychosis who have difficulty socializing [1] or to those who do not have paid employment and are therefore missing out on the contribution that work can make to self-identity and self-esteem in recovery [5,34]. While social media provides another route to online peer-to-peer support, using curated peer-based content may help in reaching groups who use social media less, such as mental health service users aged over 45 [38]. Structured peer-based content in Internet-based interventions may also appeal to young people experiencing psychosis who lack knowledge about how to search the Internet for mental health information [39] or to people experiencing schizophrenia who are interested in sharing information and emotions anonymously with peers [18].

Peers in the videos appeared to be viewed as role models by participants, similar to how peer support workers have been perceived by people in recovery [40] and consistent with psychosocial processes underlying peer support [13,35]. This finding supports further development of peer-based digital resources and research into their benefit for mental health service users. However, as noted in this study, peer-based videos are unlikely to be appealing to all service users. Walker and Bryant [40] noted that peer workers may not be viewed as role models if they are perceived to lack training or as being limited in their helpfulness by illness factors. In our study, responses to the videos appeared to be influenced by the participants’ existing ways of coping with life and managing their recovery, as well as by how they used technology. Participants who did not relate to the peers as role models perceived that their ways of coping were too different, or they were at a point in their recovery journey where they did not want to identify with peers. As Tew et al [36] argue, some people experiencing mental illness may reject being overly invested in mental health, preferring to foster broader relationships. Perceptions of technology and information on the Internet could also impact whether participants believed the lived experience videos to be trustworthy. Attention to the intrapersonal context may therefore help workers understand the choices that service users make about using digital lived experience materials. Additionally, providing a range of resources, content presented from different viewpoints, and choices about which resources to use seems important when designing future websites for this population, so that everyone can find content that matches their interests and values.

Watching the lived experience videos with a worker opened discussions about meaningful topics, thus enhancing the experience of using the website among participants in our study. The worker supported initial website use by guiding participants to navigate the website and to use the technology, when needed. One third of our participants only used SMART with the worker and close to 40% rarely or never used the Internet to access health information. Additionally, privacy is likely to be an important consideration regarding where service users choose to view videos with mental health content [41]. Thus, watching videos with a worker demonstrates potential to connect service users to peers’ experiences that they would be unlikely to access online themselves. The worker also offered choices and facilitated discussion that linked the peer videos to participants’ lives. Similarly, psychiatric nurses identified that supporting inpatients to use an educative website with peer videos facilitated discussions between them [25]. These findings provide further evidence that social presence from a clinician or researcher supports adherence to and engagement with Web-based and mobile technologies used by people with psychosis [42].

Embedding lived experience videos into service provision appears to offer a way of providing service users with hope-inspiring experiences when they meet with workers. This finding is particularly encouraging given that feeling hopeless and not getting support from their mental health services can hinder a person’s recovery [7]. Viewing and discussing the videos with a worker brought the topic of recovery to the fore in a way that would not occur if service users used the website autonomously. Hearing peers’ experiences supported participants to have confidence that their own recovery was possible and to persevere with the “hard work” of recovery [3,5]. The peer videos elicited conversations about the participant’s life experiences over time and what worked for them, rather than focusing on problems and deficits as can occur in mental health services [13]. Our focus on the experience of watching lived experience videos together contrasts with the experience of using an Internet portal for asynchronous communication [43], which was found to enrich the working relationship if service user and worker expectations about portal use were aligned, but to cause relationship tensions if expectations were not aligned. In our study, regular shared use of the website appeared to create a clear focus on recovery in interactions between the service user and worker from service users’ perspectives, potentially reducing the goal ambiguity that can characterize these relationships [30]. Together, these findings contribute to emerging evidence that using an Internet-based resource with a mental health worker, particularly a resource that includes lived experience content, can enhance mental health service provision when expectations about its use are agreed and implemented. Given the potential benefits to recovery-oriented practice, this is a strategy worthy of further investigation.

### Limitations

Limitations of this study include participants being involved in a research project, with many expressing a desire to help others experiencing mental illness through their involvement, a purpose that may influence the way that they responded in the interview [44]. Further research into using lived experience videos in other mental health practice settings would support transferability of the findings. Focusing on one element of the website, the lived experience videos, means that participants’ full use of this digital health intervention is not covered in this analysis. However, focusing on the videos, designed as a central component of the intervention, illuminated that participants experienced them as especially impactful. Understanding participants’ experience of watching videos in depth is therefore important to informing future developments in digital health interventions for people experiencing psychosis.

### Conclusions

The experience of engaging with videos featuring peers in the SMART website was powerful for participants. Hearing from peers enabled participants to feel less alone, to be inspired, and to believe that their own recovery was possible. Aspects of the intrapersonal and interpersonal contexts that existed when watching lived experience videos together shaped the way that participants perceived the experience. Contextual factors are therefore important to consider when using digital health interventions in mental health services. This study adds to emerging evidence that digital health interventions with lived experience content used by service users and mental health workers together have strong potential to support service users’ recovery and are therefore worthy of further development, implementation, and research.

## Appendix

Multimedia Appendix 1 Service user semistructured interview guide.

## Abbreviations

CGT: constructivist grounded theory
CHIME: Connectedness, Hope, Identity, Meaning, Empowerment
SMART: Self-Management And Recovery Technology
