# Supplementary material for: Recovery After Psychosis: Qualitative Study of Service User Experiences of Lived Experience Videos on a Recovery-Oriented Website
Source: JMIR Ment Health. 2018 May 8;5(2):e37. doi: 10.2196/mental.9934 (PMC5964305; doi:10.2196/mental.9934)
Supplement: Multimedia Appendix 1 [file mental_v5i2e37_app1.pdf]

Multimedia Appendix 1 Service User semi-structured interview guide.

<u>How have you used the SMART resources?</u>

Sample Prompts

- Example of a time you used SMART with therapist/mental health worker, and on your own?
- Your use of the SMART resources - where, when, who with, how often, how you accessed them; who initiated; which parts did you start with; did your use change?
- What sections of the SMART website have you tended to go back to? Feel more comfortable using? Not used very much?

<u>What was the experience like for you?</u>

Sample Prompts

- What was most helpful / least helpful; liked/disliked?
    - What was it like to use the iPad in your meetings with [add name of person]?
    - How did you find working together using an iPad?
    - How was this experience different or similar to other experiences you have of using the internet?
    - What was it like viewing videos of other people living with mental illness? Communicating with them in the forums?

<u>What made it easy to use the resources? What made it hard?</u>

Sample Prompts

- Accessing the resources when you wanted
- Ease of understanding and following instructions
- Use of peer videos, personal records, forum
- Time, interest, encouragement from others

<u>What did you get out of using SMART?</u>

Sample Prompts

- Any positive changes in you or your life since you used the SMART online resources? Any negative changes?
- Any changes to relationships with your mental health worker, family, friends, others?
- Anything unexpected or that surprised you?
- As you know, SMART stands for self-management and recovery technology. What does the term self-management mean to you? What does the term recovery mean to you? Did using SMART resources help you with these?

<u>Thinking back on your experiences of SMART and the things we have discussed today, what could be done to improve the SMART resources?</u>

<u>Is there anything else you would like to say about the SMART online resources?</u>
